# Reconstruction of large oroantral defects using a pedicled buccal fat pad

**DOI:** 10.1186/s40902-018-0144-6

**Published:** 2018-04-05

**Authors:** Sunin Yang, Yu-Jin Jee, Dong-mok Ryu

**Affiliations:** Department of Oral and Maxillofacial Surgery, Kyung Hee University Dental Hospital at Gangdong, Kyung Hee University, #892 Dongnam-ro, Gangdong-gu, Seoul, 05278 Republic of Korea

**Keywords:** Oroantral fistula, Buccal fat pad, Reconstruction

## Abstract

**Background:**

Oroantral communicating defects, characterized by a connection between the maxillary sinus and the oral cavity, are often induced by tooth extraction, removal of cysts and benign tumors, and resection of malignant tumors. The surgical defect may develop into an oroantral fistula, with resultant patient discomfort and chronic maxillary sinusitis. Small defects may close spontaneously; however, large oroantral defects generally require reconstruction. These large defects can be reconstructed with skin grafts and vascularized free flaps with or without bone graft. However, such surgical techniques are complex and technically difficult. A buccal fat pad is an effective, reliable, and straightforward material for reconstruction.

**Case presentation:**

This report describes three cases of reconstruction of large oroantral defects, all of which were covered by a pedicled buccal fat pad. Follow-up photography and radiologic imaging showed successful closure of the oroantral defects. Furthermore, there were no operative site complications, and no patient reported postsurgical discomfort.

**Conclusion:**

In conclusion, the use of the pedicled buccal fat pad is a reliable, safe, and successful method for the reconstruction of large oroantral defects.

## Background

Complete coverage of soft tissue defects is important for successful wound healing in the oral cavity. Oroantral communicating defects, which are open connections between the oral cavity and the maxillary sinus cavity, may be induced by procedures such as tooth extraction, removal of cysts and benign tumors, and resection of malignant tumors. Furthermore, treatment of cystic lesions or osteomyelitis around the maxillary sinus area can result in postoperative oroantral defects. Small defects may close spontaneously; however, large defects generally require reconstruction. Large oroantral defects may develop into oroantral fistulas or may not be covered adequately by intraoral soft tissue. Autogenous or allogenous graft materials can be used for reconstruction; however, most surgical techniques are complex and technically difficult. Among the available methods, the pedicled buccal fat pad is a simple and reliable flap for the treatment of these defects. This pedicled flap has a rich blood supply, is easily accessible, and is in close proximity to the maxillary intraoral defect [1]. The buccal fat pad flap can be an effective method for closure of small- to medium-sized oroantral communicating defects. This report represents a case series of three patients with large oroantral communicating defects that were successfully treated with pedicled buccal fat pads.

## Case presentation

### Case 1

**Fig. 1:**
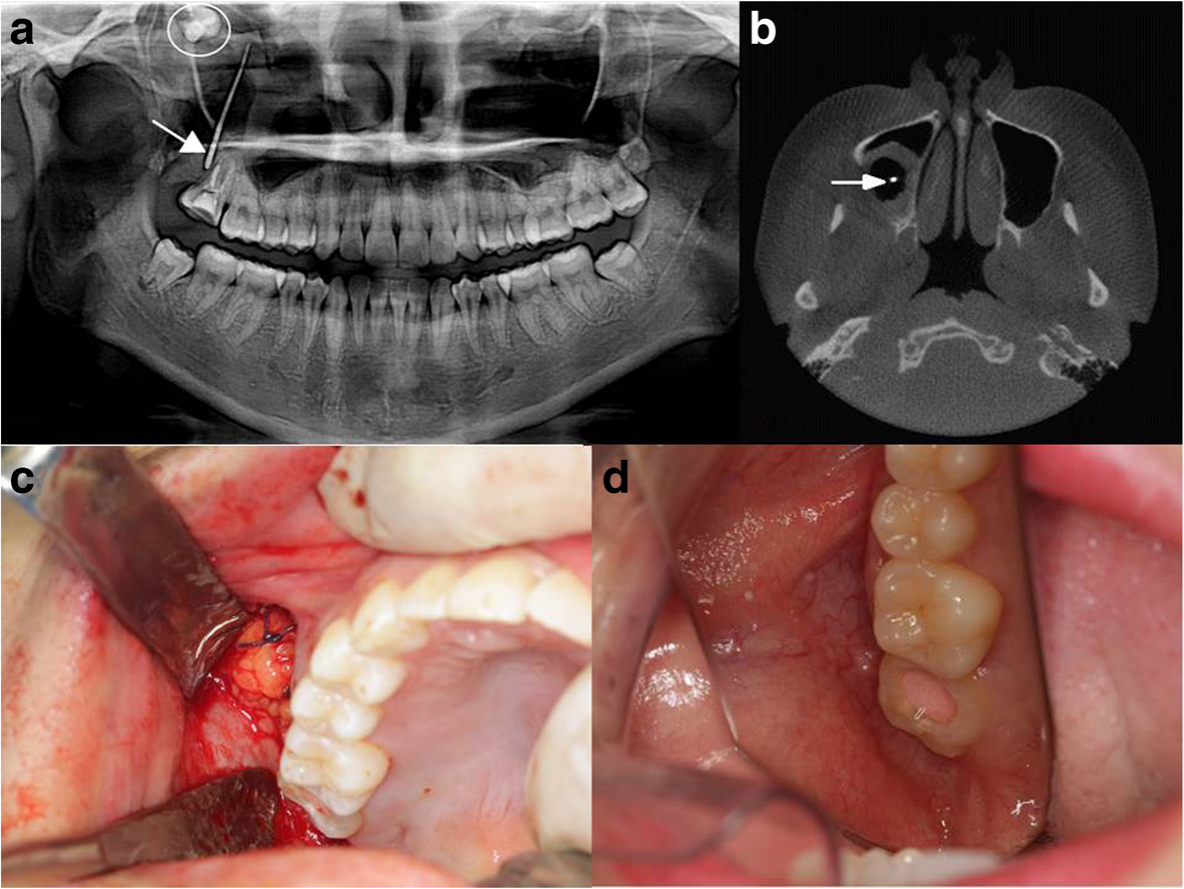
Case 1. **a** Panoramic view shows an oroantral fistula between the oral cavity and right maxillary sinus cavity (arrow indicates the gutta-percha cone). Additionally, full impaction of no. 18 was observed in the sinus cavity (circle). **b** Computed tomography showing right sinusitis and gutta-percha cone for examination (arrow). **c** The defect was reconstructed with pedicled buccal fat pad. **d** After 3 months, the operation site was successfully closed and well healed

A 21-year-old woman presented with a foul-smelling discharge and symptoms of sinusitis in the posterior area of the right maxilla. She reported intermittent discomfort in the right mid-facial area and yellowish, thick, foul-smelling pus draining from the right maxillary posterior area. Oral examination and radiologic studies revealed a chronic oroantral fistula distal to the right maxillary second molar (no. 17, Fig. 1a). A computed tomography (CT) scan showed deep, full impaction of the right maxillary third molar and a large cystic lesion (Fig. 1b). The patient underwent surgery for removal of the inflammatory tissues in the right maxillary sinus area. A cystic lesion with chronic inflammatory tissue was removed via an intraoral approach. After removal of all inflamed tissue, a large (approximately 35 mm × 20 mm) oroantral defect connecting the oral cavity and right maxillary sinus cavity was seen. A pedicled buccal fat pad was used for reconstruction of the defect (Fig. 1c). An approximately 2–3-cm mucosal incision was made below the Stensen duct. The buccinator and zygomaticus major muscles were cut, and blunt dissection was used to create an opening for herniation of the fat pad. The buccal fat pad herniated spontaneously when the superficial fascia of the face was cut. The harvested flap was placed carefully on the defect and positioned using absorbable sutures (4-0 vicryl) with minimal tension. A fissure bur was used to create a small hole on the margin of the alveolar bone to fix the pedicled fat pad in exact position. Primary closure was done on the gingival level using 3-0 black silk sutures. After surgery, the patient followed postoperative precautions under close monitoring. Three months later, clinical and radiologic examination showed good healing. There were no symptoms of right-sided chronic sinusitis or fistula, and the soft tissue had healed successfully (Fig. 1d).

### Case 2

**Fig. 2:**
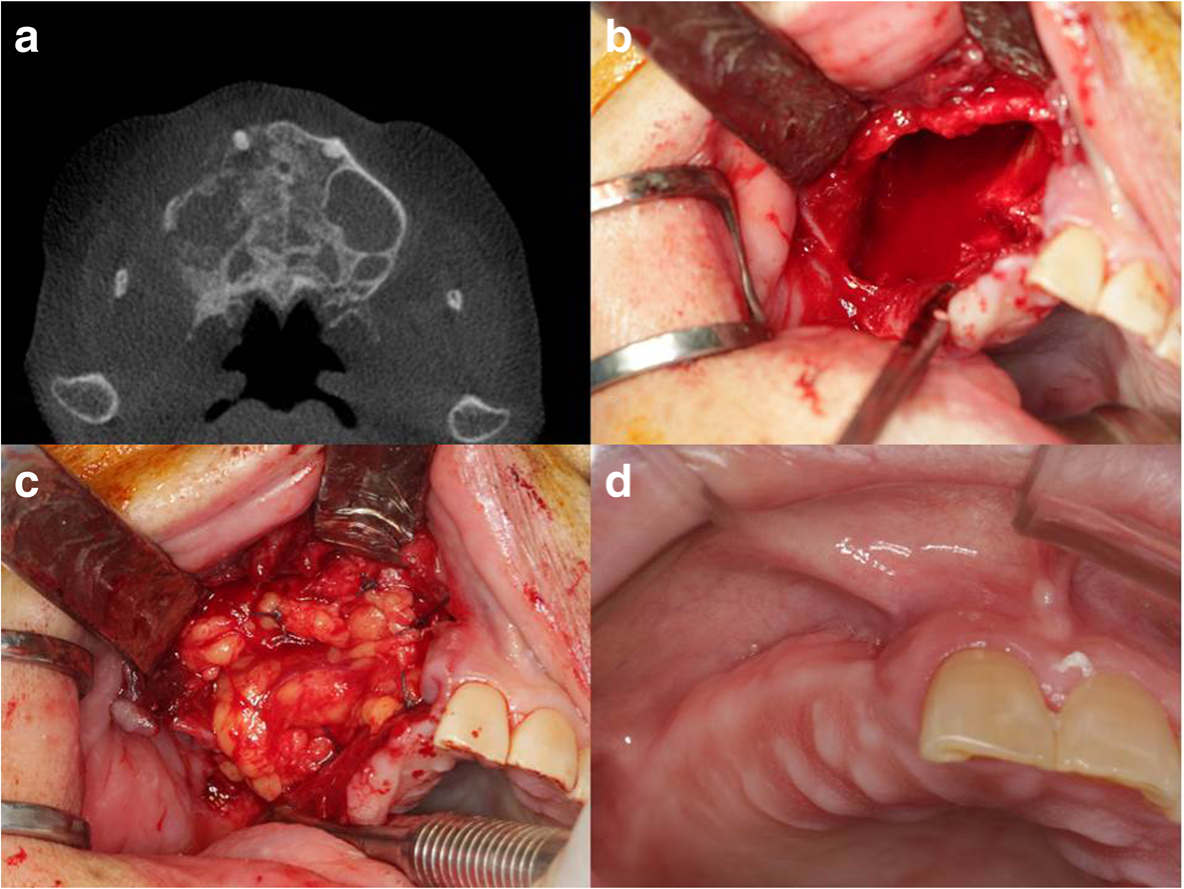
Case 2. **a** CT showed destruction of the right maxillary alveolar bone and right maxillary sinusitis. **b** Intraoperative view showed large oroantral defect after removal of inflammation tissue and sequestra. **c** The defect was covered with pedicled buccal fat pad with holding suture. **d** After 3 months, the operation site was successfully closed and well healed

An 84-year-old woman presented with right-sided, dull, facial pain. On clinical examination, pus was observed to discharge continuously from the right upper posterior area. A panoramic X-ray and a CT scan showed alveolar bone resorption and chronic inflammatory tissue in that area (Fig. 2a). The patient was diagnosed with osteomyelitis of the right maxillary posterior area, and it was decided to remove the lesion surgically. Intraoperatively, necrotic tissue, sequestra, and inflammatory tissue around the sequestrum were observed. Additionally, inflammatory tissue was present on the inferior area of the right maxillary sinus. After removal of all inflammatory tissue, a large defect (approximately 40 mm × 32 mm) connecting the oral and sinus areas remained. The defect was too large to expect spontaneous coverage by well-epithelialized soft tissue or alveolar bone. Therefore, reconstruction was performed using a right buccal fat pad as a flap (Fig. 2b). The pedicled buccal fat pad was harvested from the right-side maxilla as described in case 1. The pedicled fat pad was positioned directly on the alveolar bone using absorbable suture materials (4-0 vicryl). Primary closure was performed at the gingival level, but some exposure of buccal fat tissue remained due to excessive tension of gingival tissue (Fig. 2c). After 3 months, the patient reported no discomfort, and the site was well healed on clinical examination (Fig. 2d).

### Case 3

**Fig. 3:**
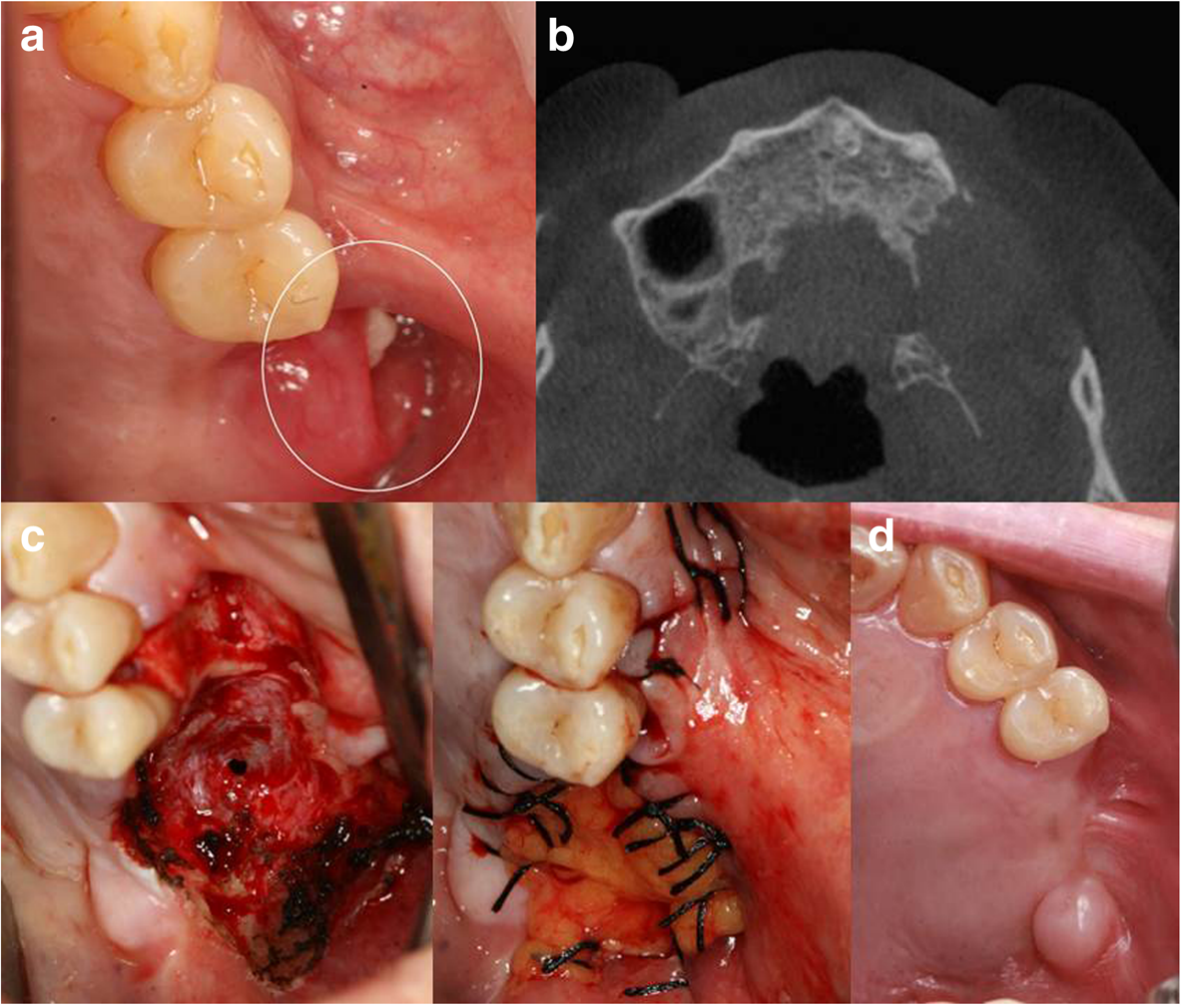
Case 3. **a** Preoperative oral examination showed a fistula on the gingiva due to recurrent ameloblastoma lesion (circle). **b** Preoperative CT showing destruction of alveolar bone on left maxillary posterior area. **c** During the surgery, a large defect was observed in the left maxillary posterior area due to the ameloblastoma. The pedicled buccal fat pad was used for coverage of the defect. The pedicled flap was exposed. **d** After 6 months, the site was well epithelized by soft tissue

An 80-year-old man presented with recurrent ameloblastoma of the left maxillary posterior area. His medical history was significant for removal of an ameloblastoma from the same area 3 years previously. Clinical examination and radiologic imaging revealed a fistula in the left maxillary posterior area due to the ameloblastoma lesion (Fig. 3a). A CT scan showed alveolar bone destruction and an ameloblastoma lesion (Fig. 3b). The patient underwent surgical removal of the ameloblastoma from the left maxillary posterior area. The ameloblastoma lesions and inflammatory tissue were removed. The defect and resorption of alveolar bone were very large (Fig. 3c). Therefore, a pedicled buccal fat pad was harvested from the left maxilla to cover the defect. A small fissure bur was used to create a hole for fixation. The flap and alveolar bone were fixed by 4-0 vicryl. There was insufficient gingival tissue for primary closure; therefore, the buccal fat pad flap and gingiva were sutured directly using 3-0 black silk. The surgical field was left with the buccal fat pad exposed. After surgery, the patient followed postoperative precautions. Five months later, clinical and radiologic examination revealed the site to be well healed, with no indications of right chronic sinusitis or oroantral fistula. Furthermore, the surgical field was well epithelialized (Fig. 3d).

## Conclusions

Oroantral communicating defects can arise secondary to dental infection, osteomyelitis, sequelae of radiation therapy, trauma, or the removal of maxillary cysts or tumors. These defects are often observed after tooth extraction in patients who have severe sinus pneumatization [2]. The extraction of maxillary posterior teeth is the most common reason of the defect because of the proximity of the apices of the bicuspids and molars to the antrum and the thinness of the antral floor (ranging from 1 to 7 mm).

The sizes of oroantral defects vary from small (1 to 2 mm in diameter) to large (over 5 mm in diameters) fistulae [2–4]. Although the size, location, and etiology differ from case to case, the soft tissue defect with difficulties in wound healing is an important feature. An oroantral defect less than 2 mm in diameter will usually close spontaneously, but when the defect exceeds 3 mm^3^, or there is inflammation in the antrum or periodontal region, the defect often persists and leads to chronic maxillary sinusitis. Various methods for closure of the defect have been reported, including a pedicled graft of the buccal fat pad [5, 6].

Since the introduction of the buccal fat pad for reconstruction of a maxillary defect in 1977 [1], many applications have been studied and introduced. A pedicled buccal fat pad can be used for epithelialization without additional skin graft procedures. Buccal fat pads have many advantages over other types of flaps [7, 8]. The surgical procedure is simple and has shown a high success rate in various applications [9, 10]. The rich vascularity of the buccal fat pad is an advantage when it is used in a poorly vascularized recipient site. Many studies have reported a high success rate (95%) using buccal fat pad procedures because of the high vascularity of the flap and its proximity to the recipient site. In addition, the surgical procedure for grafting is straightforward [11–13].

In the present cases, the large defects were successfully covered using buccal fat pads. Without the pedicled flap, large defects may not be covered by soft tissue. In case 1, primary closure of the gingiva was possible without reconstruction. However, the inner area of surgery could not be covered with well-healed epithelium without reconstruction and could not be supported by bony tissue due to resorption of bony tissue. Without reconstruction, the defect would remain and wound dehiscence would have developed. According to the follow-up examination and the radiologic data, the defect did not progress to fistula or become the source of chronic sinusitis. After the surgical reconstruction, the defect was successfully epithelized and bony defects were well covered without signs of inflammation or patient discomfort. In case 2, the defect was too large to be sutured primarily. Therefore, the pedicled buccal fat pad was used to cover the defect and primary closure was mostly achieved. At the follow-up visit after surgery, no evidence of dehiscence or recurrence was observed. In case 3, primary closure was impossible after surgical removal of a recurrent ameloblastoma lesion. Therefore, the pedicled buccal fat pad was harvested to cover the defect. The wound was exposed due to failure of gingival closure; however, the exposed fat pad was well epithelized on the operation site. The surgical wound healed perfectly without any complications. In summary, reconstruction using a pedicled buccal fat pad had the following advantages: straightforward harvest, immediate closure of the oroantral communicating defect, short surgery time, and reduced financial cost.

In conclusion, the buccal fat pad for reconstruction of large oroantral defects is a useful and straightforward alternative method for the reconstruction of various-sized surgical defects of the maxillary posterior area and even for large oroantral defects. Furthermore, reconstruction surgery using a pedicled buccal fat pad can prevent many complications that may lead to chronic inflammation of operative site and can promote epithelization of the defect site. These findings support the use of the buccal fat pad for reconstruction in the oral and maxillofacial region.

## Abbreviation

CT: Computed tomography
